# Fabrication and Characterization of a Micromachined Swirl-Shaped Ionic Polymer Metal Composite Actuator with Electrodes Exhibiting Asymmetric Resistance

**DOI:** 10.3390/s140508380

**Published:** 2014-05-12

**Authors:** Guo-Hua Feng, Kim-Min Liu

**Affiliations:** 1 Department of Mechanical Engineering, National Chung Cheng University, Chiayi 621, Taiwan; E-Mail: hahaa77@hotmail.com; 2 Advanced Institute of Manufacturing with High-Tech Innovations, National Chung Cheng University, Chiayi 621, Taiwan

**Keywords:** IPMC, Nafion, asymmetric electrode, actuator

## Abstract

This paper presents a swirl-shaped microfeatured ionic polymer-metal composite (IPMC) actuator. A novel micromachining process was developed to fabricate an array of IPMC actuators on a glass substrate and to ensure that no shortcircuits occur between the electrodes of the actuator. We demonstrated a microfluidic scheme in which surface tension was used to construct swirl-shaped planar IPMC devices of microfeature size and investigated the flow velocity of Nafion solutions, which formed the backbone polymer of the actuator, within the microchannel. The unique fabrication process yielded top and bottom electrodes that exhibited asymmetric surface resistance. A tool for measuring surface resistance was developed and used to characterize the resistances of the electrodes for the fabricated IPMC device. The actuator, which featured asymmetric electrode resistance, caused a nonzero-bias current when the device was driven using a zero-bias square wave, and we propose a circuit model to describe this phenomenon. Moreover, we discovered and characterized a bending and rotating motion when the IPMC actuator was driven using a square wave. We observed a strain rate of 14.6% and a displacement of 700 μm in the direction perpendicular to the electrode surfaces during 4.5-V actuation.

## 1. Introduction

Ionic polymer metal composites (IPMCs) are a class of electroactive polymers (EAP) that exhibit important characteristics for usage in smart sensors and actuators. IPMCs have been broadly studied as biomimetic transducers because of their large dynamic deformation in relatively low electric fields, compared with other smart materials such as piezoelectric ceramics. Nowadays, IPMCs have considerable potential as dynamic sensors, soft robots, flexible actuators and artificial muscles [1–5]. Shahinpoor and Kim published a series of review papers about IPMCs. For example, the fundamental properties and characteristics of IPMCs as biomimetic sensors and actuators and artificial muscles have been systematically presented [6]. Also, description of various techniques and experimental procedures in manufacturing ionic polymer- metal composites (IPMCs) has been reported in detail [7].

In general, an IPMC transducer consists of a backbone ionomer (e.g., Nafion) which is chemically plated metal layers (e.g., platinum) on top and bottom surfaces to serve as a pair of electrodes. The sandwiched structure allows the voltage or current which is applied the pair of electrodes to control the motion of IPMC actuator [8–12]. The working principle of IPMCs can be explained through electromechanical transduction. The bending motion is generated by the movement of the cations toward the cathode accompanied by water molecules while an electric field is exerted [13–15]. The nature of the backbone ionomer, the structure of electrodes, the cations, and the level of hydration are all significant factors that affect the performance of IPMC actuators [16].

Common researches about IPMC manufacture use a commercially available Nafion polymer sheet as a basis and grow metal electrodes on both surfaces of the sheet. A cutting tool is then employed to slice the IPMC sheet as an actuator with relative simple geometry such as a strip. Accordingly, available options for versatile IPMC actuators are restricted. Recently Feng and Chen reported the fabrication of an array of microfeatured IPMC actuators based on a silicon-bulk micromachining [17,18]. Chen and Zhang presented a photolithography method for monolithic, batch processing the Nafion sheet to form IPMC actuators which allow a complex deformation under electrical control [19]. La and Sheng applied the robust adaptive control with leakage modification to design a nonlinear controller for the IPMC's position control so the displacement response of the IPMC is stable and robust in the presence of random disturbance [20]. Besides, a micromachined 4-electrode column structured IPMC actuator enclosed a section of optical fiber, which allows the electronic directional control of conducted laser light, can be found in the literature [21].

**Figure 1.**
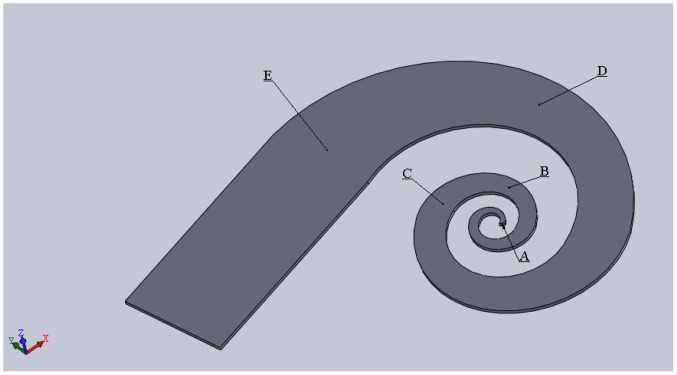
Configuration of the swirl shaped IPMC actuator with defined xyz axes.

In this paper, we propose a novel fabrication process using a microfluidic scheme to construct arbitrarily shaped planar IPMC devices. An application for the swirl-shaped IPMC could be its use as a micromirror actuator [22]. A micromirror can be placed at the center endpoint of this actuator to perform up-and-down movements for deflecting a laser beam. Besides that, the swirl-shaped IPMC can be operated in an air environment for perturbing the air flow. This could be useful for locally directing or mixing laminar gas flows. The swirl-shaped IPMC can also be applied to a biochip as a soft actuator for manipulation of bioparticles. In addition, we present study results regarding a swirl-shaped IPMC actuator fabricated using this method (Figure 1). In the proposed method, a photolithography technique was used to delineate the arbitrarily shaped IPMC actuator, enabling a swirl-shaped Nafion device to form within a photoresist-created microchannel. A sacrificial layer on a glass substrate was subsequently released, and a chemical electrode plating process was used to complete the arbitrarily shaped IPMC actuator. The design, fabrication, and device characterization are detailed in the following sections.

## 2. Device Design

**Figure 2.**
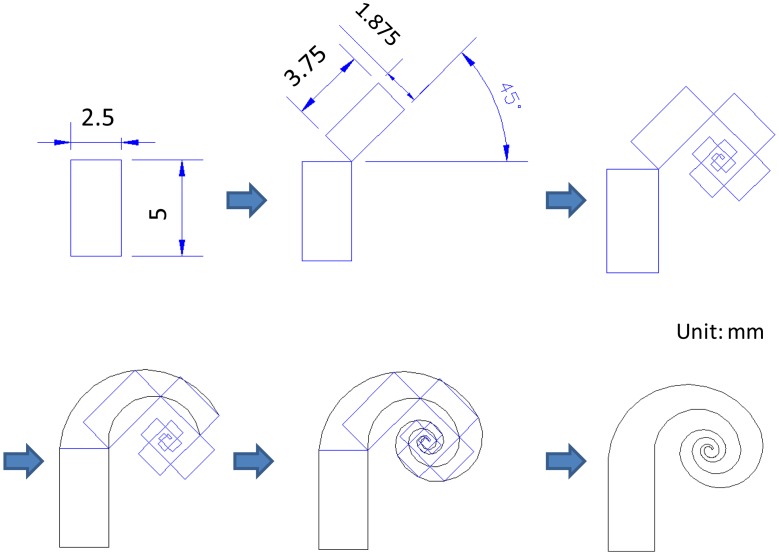
Photomask design procedure for a swirl shaped IPMC actuator.

The investigated swirl-shaped IPMC actuator was fabricated based on a photomask using a systematic design rule (Figure 2). A rectangle with dimensions of 5 mm × 2.5 mm (length-to-width ratio: 2) was first used as a reference shape. A second rectangle, featuring dimensions three-fourths those of the first rectangle (*i.e.*, 3.75 mm × 1.875 mm) but retaining the length-to-width ratio of 2, was then created. The bottom-right corner of the second rectangle was overlapped with the top-right corner of the first rectangle. The angle between the two long sides of the rectangles was set as 135°. A third rectangle was created by reducing the dimensions of the second rectangle by 75%, and the top-left corner of this rectangle was superimposed onto the top-right corner of the second rectangle. The angle formed by the long sides of the second and third rectangles was set as 90°. This process was repeated until 11 rectangles were plotted and connected. Two arcs were then drawn through the three outer and inner vertices of the first through third rectangles. The AutoCAD^®^ software function used for sketching an arc through three points was employed in this design. The remaining arcs determined according to the third through fifth, fifth through seventh, seventh through ninth, and ninth through eleventh rectangles were then drawn. The connected outer and inner curves along with the short sides of the first and eleventh rectangles construct the boundaries of the swirl-shaped actuator. The region inside the boundaries defines the microchannel used in the subsequent IPMC device fabrication. The design features a tapered width; in this study, the width was greatest (2.5 mm) at the outer end and lowest (140 μm) at the inner end.

A common strip IPMC actuator exhibits the greatest displacement at the free end, which is the farthest point from the anchor position. The purpose of the swirl-shaped design was to create an actuator that features a long moveable length at its center endpoint, which is not the farthest point from the anchor position. We investigated the electrical and mechanical properties of the swirl-shaped actuator in this study.

## 3. Device Fabrication

### 3.1. Fabrication Process

**Figure 3.**
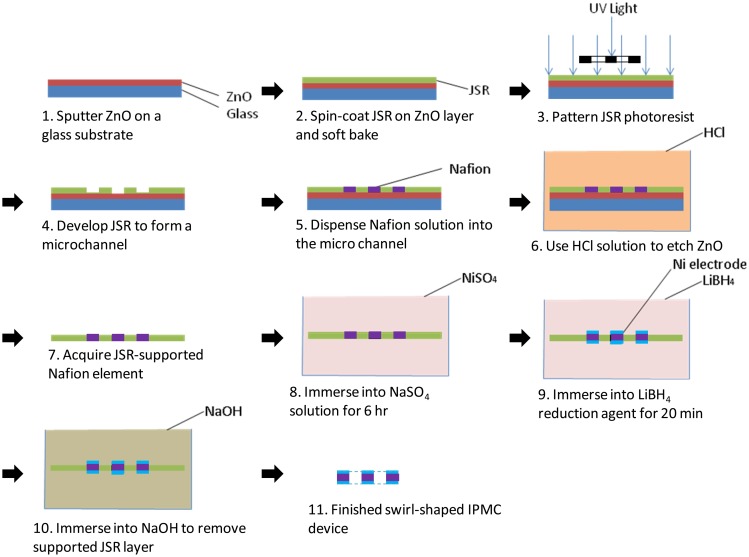
Fabrication process flow of the swirl shaped IPMC actuator.

Figure 3 shows the fabrication process flow of the developed swirl-shaped IPMC device. We began by sputtering a 2-μm-thick zinc oxide (ZnO) layer on a glass substrate. An approximately 200-μm-thick photoresist JSR was then spin coated onto the substrate. JSR is a negative-type photoresist that is suitable for shaping microfeatured structures. JSR can be dissolved in sodium hydroxide after UV exposure and baked at 150 °C; this ability is crucial for the subsequent device-releasing process [18]. The designed swirl-shaped channel was then patterned on the JSR photoresist through the photomask. The amount of 2.5 μL Nafion solution (Nafion PFSA polymer dispersions D-2021, an alcohol-based solution purchased from Dupont Co., Wilmington, DE, USA) was dropped into the fabricated microchannel in the region close to the outer endpoint by using a magnet-assisted pipette. This region, which features the greatest width in the channel, serves as a reservoir that enables the Nafion solution to be pipetted easily. Once the Nafion solution contacts the edges of the channel, the surface tension drives the Nafion solution toward the microsized portion of the channel. The characterization of the flow speed of the Nafion solution in the channel at various viscosities is detailed in the following section.

The solidification of Nafion dispersion was performed at room temperature. The Nafion solution in contact with the micromold shows hydrophilic features. We can replenish the Nafion solution through the refill region to make the surface of Nafion device nearly flat after it solidifies. During Nafion solution solidification, no cracking problem is observed with our developed fabrication method. The shrinking problem can be minimized by using our developed microfabrication technology [4]. Using the design of a refill region along with the designed shaping cavity, we replenish the Nafion solution to fully fill the designed shaping cavity through the refill region. Then, most shrinking phenomenon occurs at the refill region, which has little effect on the portion of the designed Nafion device. After the dispensed Nafion solution solidified, we immersed the processed device into a diluted hydrochloric acid (HCl) solution (H_2_O = 1:10). This treatment serves two purposes: (1) the sacrificial ZnO layer is etched to release the Nafion element along the sidewall protecting the photoresist from the glass substrate; (2) the HCl solution swells the Nafion device and exchanges the possible zinc ions inside the IPMC, produced in the solution during the ZnO layer etching, for hydrogen ions. In other words, the likely impurity ions in the IPMC could be removed by the HCl treatment.

**Figure 4.**
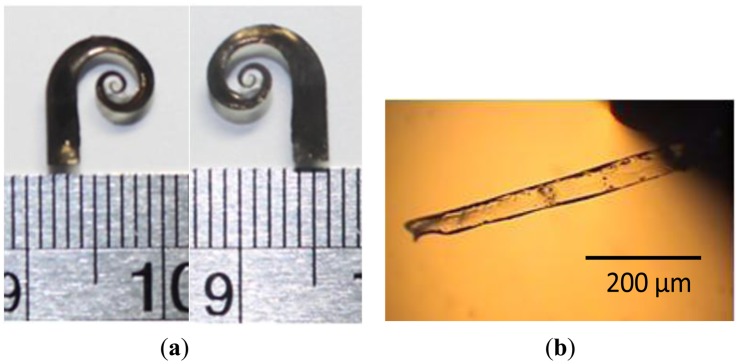
(**a**) Fabricated IPMC actuator: Top view (left) and bottom view (right); (**b**) Cross-sectional view of the IPMC actuator with a diced section.

Using nickel as the electrode could have the advantage of a high storage modulus and an increasing modulus while a magnetic field is applied. When the nickel-electroded IPMC device operates within a proper voltage range, good electric responsive properties without oxidation-induced performance degradation can be observed [23]. The released membrane, which was composed of the shaped Nafion device and protected photoresist, was soaked in a 1 M NiSO_4_ solution for 6 h. After being washed with DI water, the processed membrane was treated with a reducing agent, 1 M LiBH_4_, for 15 min to cause the nickel particles within the Nafion surface layers to precipitate on the surface of the Nafion device, thus forming the electrode. Finally, the nickel-coated membrane was immersed in a 1 M NaOH solution for 8 h. This step enables the protected JSR photoresist to dissolve in the NaOH solution. The swirl-shaped IPMC actuator was thus completed and prepared for testing (Figure 4a).

One major advantage of the proposed fabrication process is that it effectively protects the sidewalls of the microfeatured device from any metal deposition, because only the top and bottom surfaces of the IPMC device are coated with nickel electrodes. This prevents shortcircuiting during actuation. To verify the fabricated actuator without shortcircuit occurrence, we sliced a small section from the actuator and observed its cross-sectional profile under a microscope (Figure 4b). The formed Nafion layer thickness is approximately 50 μm. The transparent portion of the element represents the solidified Nafion, and the upper and lower dark edges represent the plated nickel electrodes. We confirmed that the isolation between the two electrodes was adequate.

### 3.2. Measurement of Nafion Solution Flow Speed in a Fabricated Microchannel

We observed that the Nafion solution inside the channel flowed at various velocities at room temperature for different processing periods. The experiments were performed in a laboratory environment with a relative humidity in the 30% to 70% range. The dispensed Nafion solution occasionally blocked the channel before the entire channel was filled, possibly because the solvent in Nafion solution evaporates over time, altering the evaporation rate of the solvent in Nafion solution. Therefore, a microscope equipped with a CCD camera was used to quantitatively characterize the flow velocity of the Nafion solution.

**Figure 5.**
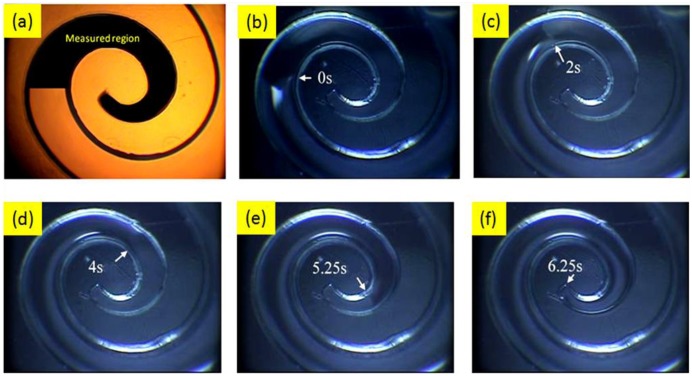
Images for characterization of Nafion solution flowing in the fabricated microchannel.

One major concern was that Nafion solution stagnated inside the channel before it was completely full. Because the Nafion solution flowed from the outer endpoint to the inner endpoint of the designed swirl-shaped channel, we focused on the channel region near the endpoint to observe details. Figure 5a shows the image captured using the microscope with a field of view set around the central portion of the channel. The flow velocity characterization was focused on the black marked region. The measurement procedure is described as follows. We slowly pipetted the defined amount of Nafion solution into the reservoir region of the channel. The Nafion solution flowed along the channel, driven by the surface tension induced by the side and bottom walls. Figure 5b–f shows images captured at various time intervals. The travel angles of the flow contacting the inner and outer walls were similar, indicating that the flow close to the outer wall was faster than that close to the inner wall. Because the channel was not constructed of a simple geometric shape, such as a circle, we measured regions with the same inner and outer radii. Therefore, the distance *D* between two specific points on the sidewalls could be calculated according to the equation *D* = *R* × *θ*, where *R* is the radius and *θ* is the angle between the two points. We measured the travel distance from the point at which the flow entered the black region to the point at which the flow reached the center endpoint of the swirl-shaped channel.

**Figure 6.**
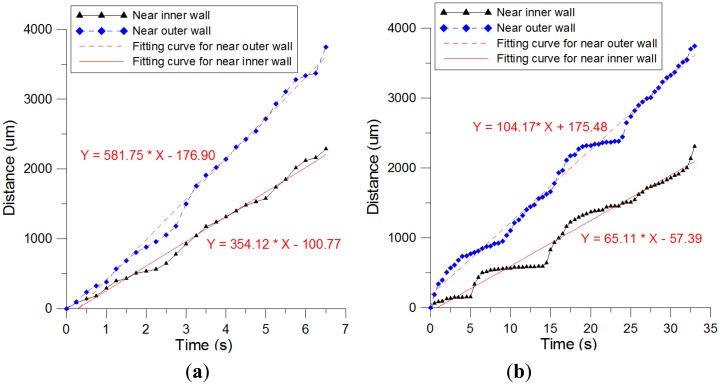
Results of Nafion solution flow velocity measurement: processed at (**a**) 5 min (**b**) 1 h after taken from refrigerator.

We compared the images captured by the camera at various time intervals and identified the locations the Nafion flow front reached. Figure 6a shows the relationship between the flow distance and flow time when the Nafion solution was removed from a −5 °C refrigerator, kept at room temperature (25 °C) for 5 min, and then dispensed into a pipette. The results obtained when the Nafion solution was kept at room temperature for 1 h and then distributed into the channel reservoir are shown in Figure 6b. We observed some stagnation effects at certain locations, and the time required for the Nafion solution to reach the endpoint was greater than 30 s. The slopes of the fitting curves represent approximate average flow velocities. The results revealed that the flow velocity measured after the Nafion solution was exposed to room temperature for 60 min was 20% of that measured after the solution was kept at room temperature for 5 min. However, the Nafion solution still filled the channel without creating a void. Since the Nafion solution containing 34% water and 44% 1-propanol which is easily vaporized in an opened container at room temperature, the reduced amount of water and 1-propanol decreases the mobility of Nafion. Thus, the Nafion taken out 5 min from the fridge and kept at room temperature has faster flow-rate than the Nafion kept at room temperature for 1 h.

### 3.3. Surface Roughness Measurement of the Processed Nafion Device

In the proposed process, the top surface of the solidified Nafion device formed through the evaporation of the solvent in Nafion solution inside an open channel (*i.e.*, the surface was simply interfaced with the air). However, the bottom surface was interfaced with the ZnO layer coated on the glass substrate. This asymmetric surface formation condition yielded dissimilar roughness on both surfaces and affected subsequent metal electrode growth.

**Figure 7.**
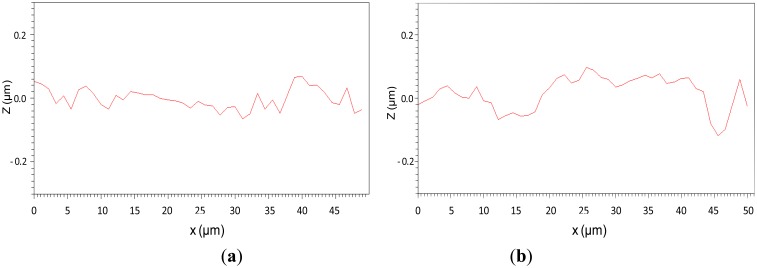
Surface roughness of (**a**) front surface (**b**) bottom surface of the fabricated Nafion element.

Figure 7 shows the results of the surface roughness measurements obtained using a white-light interferometer (BMT Co., Tuttlingen, Germany). The amplitude of fluctuation on the top surface was approximately 0.1 μm, and that on the bottom surface was 0.25 μm. A sputtered ZnO film typically exhibits a rough surface. This surface effect substantially increased the degree of roughness at the interface between the ZnO film and solidified Nafion.

## 4. Experimental Setup

### 4.1. Resistance Measurement of the Surface Electrodes of the Fabricated Actuator

**Figure 8.**
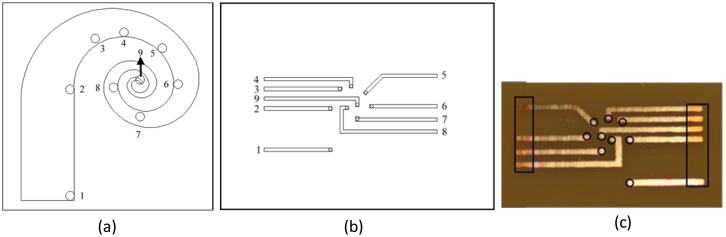
(**a**) Nine points selected for surface resistance measurement; (**b**) Illustration of the developed tool for surface resistance measurement; (**c**) Implementation of the tool on a PCB chip.

Because the top and bottom electrodes were grown under the different interface conditions described in the Device Fabrication section, and the swirl-shaped geometry and tapered width were unique, we investigated the surface resistance at various locations on the device. The measurement was not trivial because the finished IPMC actuator was constructed of a soft material; using probes to gauge the resistance and maintain equal pressure on the detected points on the device is essential. We implemented a resistance detection device by using a piece of print circuit board (PCB). In Figure 8a, the measured locations are numbered. These locations were contacted with conductive patterns on the PCB, and the outer ends of the patterns were then soldered to wires to facilitate measurement using an LCR meter.

The layout of the photomask used in fabricating conductive patterns on the PCB is shown in Figure 8b. Standard photolithography and etching processes were used to engrave the conductive pattern on the PCB (Figure 8c). Because of the swirl-shaped design, the conductive patterns crossing multiple points of the fabricated actuator might hinder resistance measurement. An isolation layer was deposited to solve the problem. A 2-μm-thick pinhole-free, conformal parylene was coated on the processed PCB. The photoresist AE 5214 was then applied to the processed PCB. Nine circular holes were patterned for use in resistance measurement, as shown in Figure 8b. The oxygen plasma reactive ion etching process was performed to remove the parylene on specific regions. The PCB, which featured conductive patterns and a selective area isolated by parylene, was thus processed.

To facilitate surface resistance measurement, the outer conductive patterns without parylene were soldered to wires so that they could easily be connected with the LCR meter. The swirl-shaped IPMC actuator was aligned and positioned on the fabricated PCB device. A glass slide was diced to an appropriate size to fully cover the IPMC actuator but not the wire soldering region. The diced glass slide was then placed on the IPMC actuator, and a 50-g weight was placed on top of the glass slide. The glass slide and additional weight provided suitable pressure between the IPMC actuator and the conductive patterns at the measured locations. We then recorded the resistance values between various detection points of the electrodes on both sides of the IPMC actuator.

### 4.2. Characterization of the Driving Voltage and Current of the Fabricated Device

**Figure 9.**
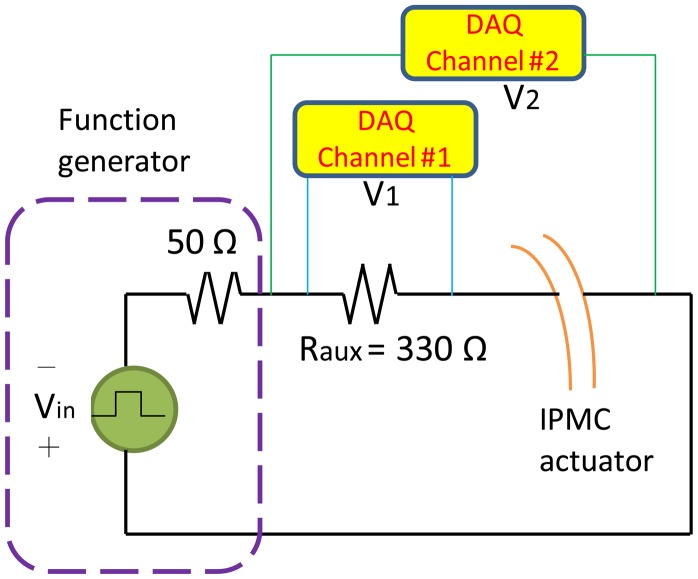
Designed circuitry for measuring the voltage across and current through the IPMC actuator.

To characterize the electrical properties of the IPMC actuator, the wide-ended portion of the actuator was anchored to an insulating clip by using two strips of conductive tape, which were individually adhered to each side of the clip as electrode pads. Control signals were applied to both electrodes to activate the IPMC actuator. A circuitry designed for acquiring the voltage drop between the electrodes and the current flowing through the actuator was used (Figure 9). A 330-Ω resistor (*R_aux_*) served as an auxiliary element. A function generator drove the device by producing a square waveform with zero bias. Driving signals with differing amplitudes operating at a frequency of 0.1 Hz were studied and compared. The voltage drops across the auxiliary resistor *V*_1_(*t*) and across both the resistor and IPMC actuator *V_2_*(*t*) were acquired using a data acquisition system (Model #6211, National Instrument, Austin, TX, USA). Because the internal resistance (>1 GΩ) of the data acquisition system was much greater than the auxiliary resistor resistance, the current flowing through the data acquisition system was ignored. Therefore, the current flowing through the IPMC device was calculated according to the following equation: *id*(*t*) = *V_1_*(*t*)/*R_aux_*. The voltage drop across the device was calculated according to the following equation: *V_d_*(*t*) = *V_2_*(*t*) − *V_1_*(*t*).

### 4.3. Multipoint Displacement Measurement of the Fabricated Device

A CMOS video camera was employed in capturing images continually at a frame rate of 30 fps to track the displacement of the device. However, characterizing the displacement of the fabricated device is not as simple as characterizing that of a one-dimensional device such as an IPMC strip, because the swirl-shaped planar structure can consist of multidimensional displacements. To investigate this phenomenon, two cameras were set at different view angles. The optical axis of the first camera was perpendicular to the horizontally placed IPMC actuator, thus enabling the in-plane motion (*xy* direction Figure 1) to be monitored. The optical axis of the second camera was set at a 45° angle, crossing that of the first camera. Because the distance between the device and the camera was much greater than the displacement of the device, the principle of triangular geometry was applied to acquire the *z*-axis displacement from captured images. The five points indicated in Figure 1 were used to characterize the displacement. The captured images were processed using AutoCAD^®^ software to determine the displacement at selected points according to a calibrated scale.

## 5. Results and Discussion

### 5.1. Results of Surface Resistance Measurement and the Electrical Actuation Property of the Fabricated IPMC Actuator

**Figure 10.**
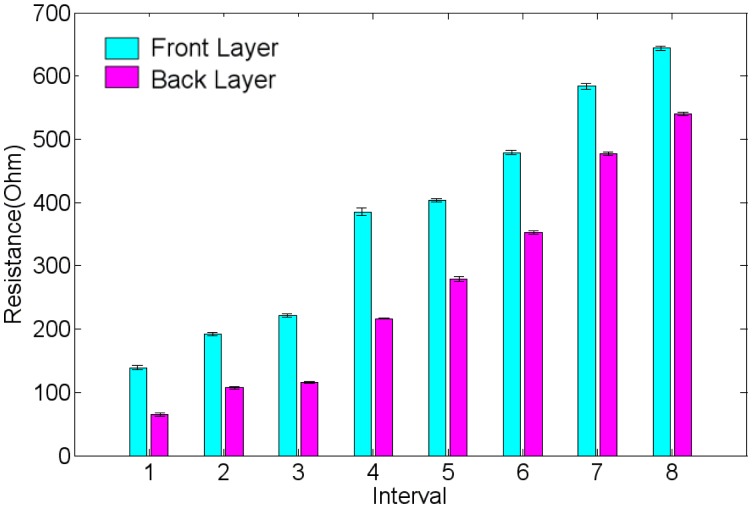
Measured surface resistances of the fabricated swirl shaped IPMC actuator.

Figure 10 shows the measured results of surface resistance for the top and bottom electrodes of the fabricated IPMC actuator. The numbers 1 through 8 shown on the horizontal axis represent the resistance values between the defined locations 1 and 2, those between 1 and 3, and so on (Figure 8a). For both electrodes, the longer the measured length of the actuator portion was, the higher the resistance value that could be obtained. Comparing each measured interval for both electrodes indicated that the top electrode, which formed on the shaped Nafion surface interfaced with the air, always exhibited a higher resistance than did the bottom electrode, which formed on the shaped Nafion surface interfaced with the sputtered ZnO layer. The difference in resistance from 80 to 150 Ω can be attributed to the surface roughness of the shaped Nafion device, as described in the Device Fabrication section. The resistance asymmetry between electrodes causes asymmetrical motion during actuation, as detailed in the subsequent section.

**Figure 11.**
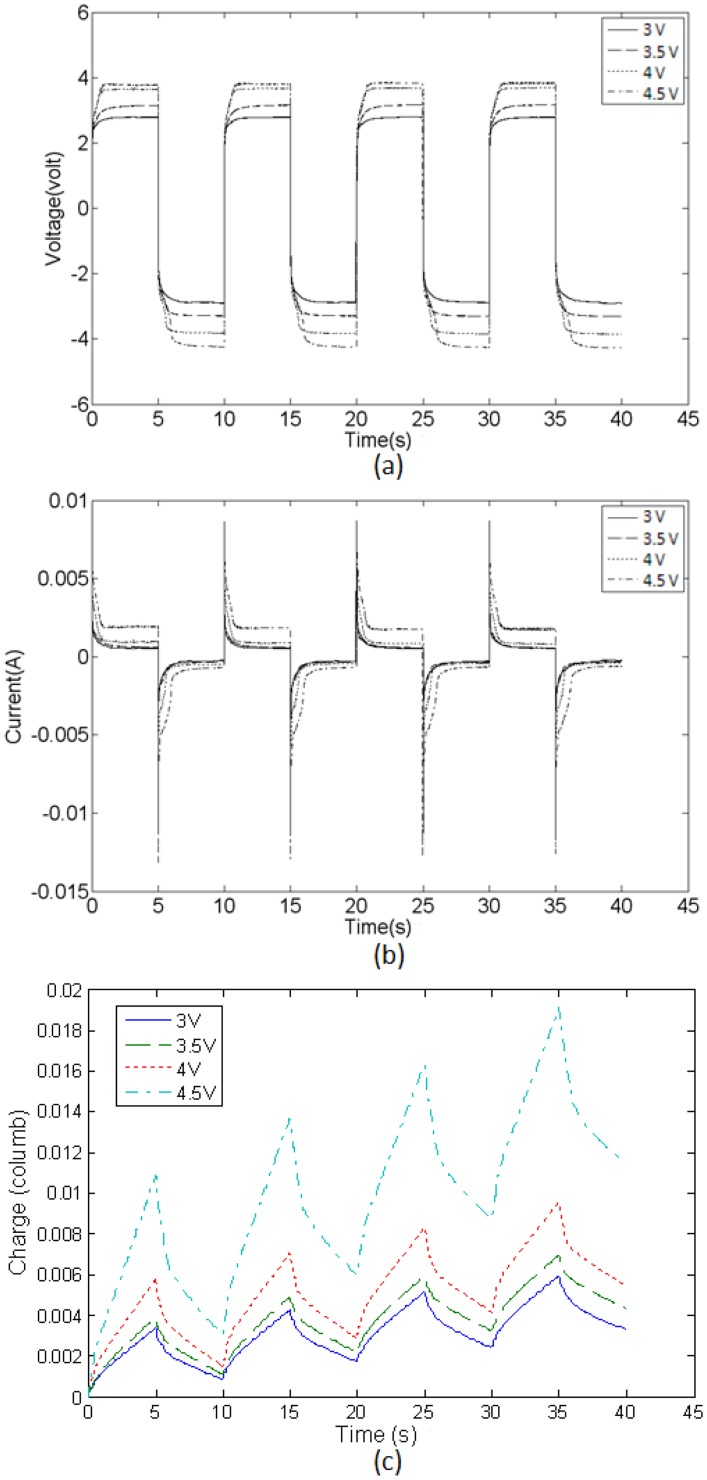
(**a**,**b**) The voltage across and current flowing through the top and bottom electrodes measured at the anchor location of the IPMC actuator; (**c**) The corresponding charge caused by current flow.

Figure 11 shows the voltage across and current flowing through the top and bottom electrodes measured at the anchor location of the IPMC actuator. The driving signals were bipolar square waveforms with amplitudes of 3, 3.5, 4, and 4.5 V. During the first half of the cycle, positive voltage was applied to the top electrode, and the current flowed from the top electrode to the bottom electrode. During the second half of the cycle, negative voltage was applied to the top electrode, and the current flowed from the bottom electrode to the top electrode (negative current values). The resulting voltages rapidly reached approximately constant values within 1 s. The current exhibited an obvious charging effect attributable to the capacitance effect of the IPMC device. The current curve indicated an asymmetric phenomenon about zero current values for all cases. In this study, nickel is used as an electrode material and it would be not so stable as gold or platinum. For the driving case of 4.5 V, the current through the actuator increases dramatically compared with other driving cases. This could be explained as Faradic process occurrence while the applied voltage reaches 4.5 V (Figure 11b).

**Figure 12.**
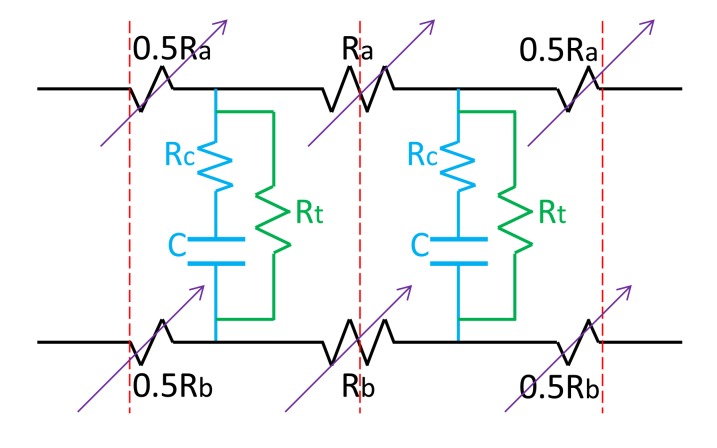
Circuit model for our swirl shaped IPMC actuator using presented fabrication process.

To describe the electrical characteristics of the fabricated IPMC actuator, we used a circuit model reported in the literature [24] (Figure 12). This model can be used to delineate the asymmetric surface resistance characteristics of the top and bottom electrodes. In this model, we use the variable resistor R_a_ and R_b_ to indicate the surface resistance of the top and bottom electrodes, respectively, and R_t_ represents the “through-polymer” resistance. The capacitance C in conjunction with resistor R_c_ can be considered the simplified impedance for ion movement dynamics, representing the characteristics of the exponential step response curve of the current. The values of the variable resistors R_a_ and R_b_ rely on the curvature/displacement of the IPMC actuator at the given point [24]. When the surface stretches or compresses the R_a_ and R_b_ varies differently. Thus, our experimental results can be justified with this circuit model.

### 5.2. Results of Multipoint Displacement Measurement of the Fabricated Device

**Figure 13.**
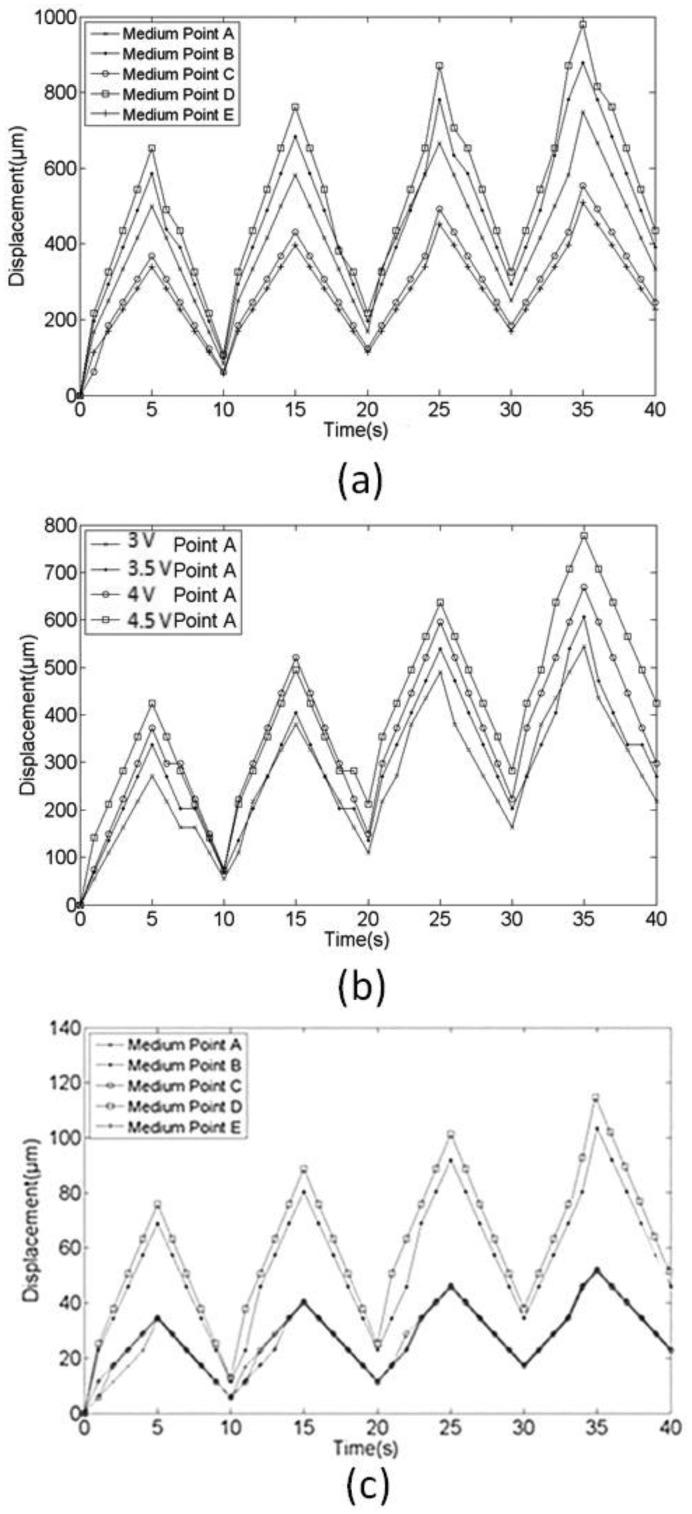
(**a**) Displacements at five selected points under a 0.1 Hz 4.5 V bipolar square wave actuation; (**b**) Displacement result in the z direction at the inner endpoint A of the IPMC actuator based on different external actuation voltages; (**c**) displacements in *x*-direction for five selected locations based on 4.5 V actuation.

Figure 13a shows the results regarding periodic displacement in the z direction (Figure 1) at the five selected points when 0.1-Hz, 4.5-V bipolar square wave actuation was applied. We observed the highest value at Point D, which is the point on the IPMC actuator farthest from the anchor in the defined *x* direction. The points are ranked according to displacement in the *x* and *y* directions, respectively, as follows: D > B > A and A > C > E. The upward displacements (*i.e.*, the current flow from the top to the bottom electrodes) exhibited relatively high values compared with the downward displacements at all selected points. Hence, non-horizontal baselines were observed according to all measured displacement data. This can be attributed to the generated unbalanced strains of the IPMC actuator caused by the driving currents during upward and downward motions. The fabricated IPMC can be operated in air for approximately 5 min before the need for re-hydration. In addition, the total charge *Q* of the swirl-shaped IPMC actuator on the surface at time t can be expressed as:

(1) Q(t)=∫i(t)dt

The term *i(t)* is the current flowing through the actuator and can be obtained experimentally.

According to our current measurement, the charge calculation for each driving cycle can be expressed as shown in Figure 11c. The unequal distribution of charge magnitudes in each half cycle indicated the different slopes of upward and downward moment generation, so as the displacement. Thus, the phenomenon of the nonzero-slope baseline for the measured displacement was justified.

Furthermore, we defined the *x*-direction strain ratio as the *z*-directional displacement divided by the *x* coordinates of the five selected points (the anchor position *x* coordinate is set to zero), and the results are listed in Table 1. The maximal value was observed at the inner endpoint. Location D shows the turning point of this ratio. A length of the actuator greater or less than this point exhibited an increased ratio.

Figure 13b shows the displacement result at the inner endpoint, Point A, of the IPMC actuator according to various external actuation voltages. A higher voltage causes greater displacement, but a nonlinear trend can be observed. We examined the steady-state current flowing through the actuator in the case in which the device was driven at 4.5 V, and the current exhibited a pattern different from those of the other three voltage actuation cases, particularly during the upward motion. Figure 13c shows the displacements in *x*-direction for five selected locations. The values are much smaller than the *z*-directional displacements. The points with larger *x*-coordinates demonstrate larger displacement values. Points A, C, and E have almost similar displacements. Since the displacement in *y*-direction is even smaller than in *x* direction; therefore, we determined that the motion of the fabricated swirl-shaped actuator IPMC was substantial in the *z* direction and slight in the *x* direction.

Table 2 compares existing IPMC devices and the swirl-shaped IPMC actuator in terms of the raw material, shaping method, possibility of fabrication with microfeatures, complex geometry of devices and their functions. The results indicate our developed fabrication method, along with the presented device, have unique characteristics.

## 6. Conclusions

We have designed, fabricated, and tested a micromachined swirl-shaped IPMC actuator. The microfeatured IPMC device fabricated using the proposed process features plated metal layers only at the top and bottom regions and does not contain sidewalls, preventing the occurrence of shortcircuits. Conductive patterns were created on a PCB chip serving as a surface resistance measurement tool for the fabricated IPMC actuator. The top and bottom electrodes exhibited differences in surface resistance ranging from 80 to 150 Ω. The bottom electrodes consistently exhibited surface resistance values lower than those of the top electrodes and this could be due to the device fabrication process with rough rough-surfaced ZnO layer as a sacrificial layer. The current flow patterns exhibit asymmetric in relation to zero current values when bipolar square waves were used to drive the actuator with zero voltage bias. The motion of the fabricated actuator was characterized by measuring five selected points on the device. The results indicated that the actuator produced substantial out-of-plane displacement (the direction perpendicular to both electrodes) and slight in-plane displacement. The developed microfabrication process enables the creation of a versatile microfeatured IPMC actuator. The swirl-shaped actuator exhibited a simultaneous bending and rotating motion and might be useful as an active reflector for directing electromagnetic waves or laser beams.

## Figures and Tables

**Table 1.** *x*-direction strain ratio defined as the *z*-direction displacement divided by the *x* coordinates of the five selected points.

**Location**	***x* Coordinate (mm)**	**Strain Ratio**
A	3.42	14.59%
B	4.51	12.98%
C	3.25	11.35%
D	6.96	9.38%
E	3	11.31%

**Table 2.** Comparison and summary of existing and our developed IPMC devices.

**Ref.**	**Raw Material**	**Shaping Method**	**Possibility for Fabricating Microfeatures**	**Fabricating Complex Geometry**	**Device Function**
[25]	Nafion film	Knife cutting	Depending on cutting skill	Depending on cutting skill	Biomimetic robotic Venus flytrap
[26]	Nafion film	Cutting and thermal treatment	Sliced strip determines the feature	Suitable for making helical structure	Helical IPMC actuators for radius control
[19]	Nafion film	Knife cutting	Electrode can be made with microfeatures	Photolithography-made electrodes can have complex geometry	Monolithic fabrication of multi degree-of-freedom IPMC actuator
[27]	Nafion film	Laser machining	Yes (depend on laser spots)	Controlled by moving work station	Microstrips for microsensing and microactuation
This work	Nafion solution	Micromold using photolithography	Yes (microfabrication technology)	Depending on the microchannel design	Swirl-shaped device for directing laser beams
